# Identification and Characterization of Glycine Decarboxylase as a Direct Target of Snail in the Epithelial–Mesenchymal Transition of Cancer Cells

**DOI:** 10.4103/tme.tme_8_18

**Published:** 2019-02-04

**Authors:** Guohua Chen, Jianmei Wu, Jing Li, Jian Wang

**Affiliations:** 1 Department of Pathology, Wayne State University School of Medicine, Detroit, MI, USA; 2 Karmanos Cancer Institute, Wayne State University School of Medicine, Detroit, MI, USA; 3 Cardiovascular Research Institute, Wayne State University School of Medicine, Detroit, MI, USA

**Keywords:** Cancer metabolism, epithelial–mesenchymal transition, glycine catabolism, glycine decarboxylase, Snail

## Abstract

**Context/Aims:**

Metabolic reprogramming and cellular plasticity drive tumorigenesis. However, how these cellular events collectively contribute to the oncogenic process is poorly understood. Epithelial-mesenchymal transition (EMT), a fundamental mechanism of cellular plasticity, is governed by the EMT transcription repressors such as Snail. In the present study, through establishment and characterization of inducible overexpression of Snail in A549 lung cancer cells, we aim to define the metabolic reprogramming in response to Snail in the EMT of lung cancer cells.

**Methods/Results:**

Our metabolomic analysis suggests that forced expression of Snail accompanied reduced diversion of glycolytic metabolites to the serine/glycine metabolic shunt, a critical metabolic branch that distributes glucose catabolic intermediates to the major anabolic pathways. Our gene expression profiling and molecular characterization revealed that Snail actively suppressed the expression of glycine decarboxylase (GLDC), a key enzyme on the serine/glycine metabolic shunt, through binding to an evolutionarily conserved E-box motif and thereby inhibiting the promoter of the GLDC gene. Besides, knockdown of GLDC led to a cellular function shift from proliferation to migration.

**Conclusion:**

This study has revealed a novel molecular link that integrates the serine/glycine metabolism with the Snail-mediated EMT program in cancer cells.

## Introduction

During embryogenesis, multiple waves of transdifferentiation from the epithelial to the mesenchymal cell type denoted as the epithelial–mesenchymal transition (EMT), occur in the developing embryo, which allows the primitive embryonic cells to migrate and form the three germ layers fundamental to the development of a multicellular organism, including human beings.^[1,2]^ It is increasingly clear that tumor cells are able to hijack the capacities of EMT to achieve metastasis and chemoresistance.^[3,4]^ At the molecular level, EMT transcription factors such as Snail govern the occurrence of EMT through binding to the E-box regulatory motif (s) and thereby repressing the key epithelial genes such as E-cadherin.^[5,6]^

Interestingly, while gaining migratory capacity during EMT, the cells often slow down proliferation, implying that significant rearrangement of cellular states including cellular metabolism would occur in the EMT processes.^[7–9]^ However, the metabolic phenotype associated with the EMT of cancer cells is poorly defined and appears to be context dependent. For example, the elevation of Snail has been shown to augment differential responses in the glycolysis, the primary catabolic pathway of cancer cells,^[10]^ with the upregulated and downregulated glycolysis seen in the breast^[11]^ and lung cancer,^[12]^ respectively. Therefore, it is imperative to elucidate the molecular relationship between the EMT transcription factor and the metabolic target for a better understanding of the metabolic basis for EMT.

The small amino acid glycine is a key metabolic intermediate that connects the major catabolic and anabolic pathways in cancer cells.^[13,14]^ Synthesized from the serine that is derived from the glycolytic catabolites, the glycine is broken down to form the folate-mediated one-carbon unit, supplying key anabolic intermediates for supporting cell proliferation.^[13,14]^ Therefore, high turnover of glycine is a unique metabolic feature associated with rapidly proliferating cancer^[15]^ and embryonic stem cells.^[16]^ Consistently, the key enzymes of this high-flux serine/glycine metabolic shunt including phosphoglycerate dehydrogenase, serine hydroxymethyltransferase, and glycine decarboxylase (GLDC) which are responsible for the synthesis and catabolism of serine and glycine, respectively, are all found to be significantly elevated to drive cancer proliferation.^[17–19]^ However, given a clear functional shift from proliferation to migration during EMT, it is not known whether the serine/glycine metabolic shunt is reprogrammed at the molecular level in this important cellular process.

In the current study, through characterization of an EMT tissue culture model, we identified that GLDC, the glycine catabolic enzyme is a novel direct target of Snail. We found that Snail binds to an evolutionarily conserved proximal E-box motif in the *GLDC* gene and thereby suppresses the *GLDC* promoter. Thus, these observations have revealed a novel molecular link that integrates the serine/glycine metabolic rearrangement with the Snail-induced EMT program.

## Materials and Methods

### Cell culture, plasmid construction, and cell line establishment.

Human lung cancer A549 and H1299 (ATCC) and human embryonic kidney 293FT (Invitrogen) cells were maintained in Dulbecco’s modified Eagle medium supplemented with 10% fetal bovine serum, 100 U of penicillin/ml, and 0.1 ng of streptomycin/ml.

For construction of the Snail expression vectors, the full-length human Snail cDNA was fused with a 3’ in-frame HA epitope tag sequence and was cloned into pInducer20^[20]^ and pcDNA_3.1_ (Invitrogen) to yield pInducer20-Snail-HA and pcDNA_3.1_-Snail-HA for doxycycline-inducible and constitutive overexpression of wild-type Snail, respectively. For construction of the expression vector for the transcriptionally inactive Snail mutant that lacks SNAG domain,^[21]^ the sequence encoding the amino acids at the positions of 2–8 at the N-terminal of Snail protein, was deleted in pcDNA_3.1_-Snail-HA to yield pcDNA_3.1_-Snail_ΔSNAG_-HA using a site-directed mutagenesis kit (Stratagene). For construction of the luciferase reporter for *CDH1* promoter, a ~400 bp sequence spanning the nucleotide positions-365 to +48 of *CDH1* gene was amplified from human genomic DNA and cloned into pGL3-basic (Promega). For construction of the luciferase reporters for *GLDC* promoter, a ~1 kb sequence spanning the nucleotide positions-962 to +55 of *GLDC* gene was amplified from human genomic DNA and cloned into pGL3-basic (Promega) to yield the wild-type reporter GLDC-luc. The mutated reporter constructs with targeted deletion of either the proximal or the distal E-box motif were generated using site-directed mutagenesis and designated as E1 mut-luc and E2 mut-luc, respectively.

For establishment of A549-Snail cell line, the lentiviral particles were produced by cotransfection of pInducer20-Snail-HA with pMD2.G and psPAX2 packaging vectors into 293FT cells, and were then infected into A549 cells. The infectants were selected in the media containing 500 μg/ml of G418, and the drug-resistant cell clones were screened for doxycycline-inducible overexpression of Snail-HA protein using Western blotting.

For knockdown of GLDC, the siRNA oligos (Origene) was transfected into A549 cells using Lipofectamine RNAiMAX reagent according to the vendor’s specifications.

### Western blotting

Cells were washed twice in phosphate-buffered saline (PBS) and lysed in the hypotonic lysis buffer (25 mM Tris-HCl-pH 7.5, 40 mM NaCl, 1% Triton X-100). Protein concentrations were determined with the Bradford reagent (Bio-Rad). Cell lysates (40 μg) were resolved by sodium dodecyl sulfate-polyacrylamide gel electrophoresis, and proteins were transferred onto nitrocellulose filters. The blots were saturated with 5% nonfat milk and probed with antibodies against GLDC (Sigma, 1:1000), AMT (Sigma, 1:1000), DLD (Sigma, 1:1000), GAPDH (Santa Cruz, 1:1000), E-Cadherin (BD Bioscience, 1:1000), N-Cadherin (Abcam, 1:1000), Snail (Cell Signaling, 1:1000), or HA (Santa Cruz, 1:1000). Following a wash with PBST (PBS containing 0.1% Tween 20), the blots were incubated with peroxidase-coupled goat anti-rabbit immunoglobulin G (Sigma, 1:5000). The immunolabeled protein bands were detected by enhanced chemiluminescence method (Perkin Elmer).

### Immunohistochemistry

Cells grown in the chamber slides were fixated with 4% paraformaldehyde, and subsequently permeabilized with 0.2% Triton X-100. After saturation with 3% Donkey serum, cells were incubated with the primary antibody against HA (Santa Cruz, 1:250) or E-Cadherin (BD Bioscience, 1:250), and then incubated with the TRITC-conjugated Donkey anti-rabbit (Jackson ImmunoResearch, 1:500) or FITC-conjugated Donkey anti-mouse (Jackson ImmunoResearch, 1:500) secondary antibody. The immunofluorescence was visualized in the cells using confocal microscopy (Olympus).

### Cell proliferation and migration assays

Cell proliferation and migration were examined as previously described.^[22,23]^ To measure cell proliferation, equal number of cells were seeded on 6-cm plates, and were subjected the indicated treatment. The number of cells was then counted using a hemocytometer at the indicated time. To measure cellular capacity for migration, the Boyden chamber matrigel migration assay was performed. In brief, cells were seeded in the serum-free media in 8 μm Boyden chamber (Corning) coated with Matrigel (BD Bioscience), and the chamber was placed inside a bottom plate containing complete growth media as migratory attractants. Following 24 h incubation, invasive cells retained on the filter were fixed with 1% paraformaldehyde and stained with 1% crystal violet, and quantified under a microscope.

### Quantitation of intracellular metabolites

Cell metabolism was quenched by flash freezing of the cells in liquid nitrogen. Cellular metabolites were extracted in 80% aqueous methanol, and then quantitated using the LC-MS/MS methods as previously described.^[24]^ Data are presented as the relative abundance of each individual metabolite in reference to the level of the control cells without Snail induction.

### Determination of mRNA expression by next‑generation sequencing and quantitative reverse transcription‑polymerase chain reaction

Total cellular RNA was isolated using the Trizol reagent (Invitrogen). To profile the differential expression of mRNAs in the A549-Snail cells with or without Snail induction, cDNA libraries compatible for Illumina sequencing were prepared by using the QuantSeq 3’ mRNA-seq Reverse (REV) Library Prep Kit (Lexogen) according to the manufacturer’s instruction. The resultant cDNA libraries were assessed using a TapeStation (Agilent), and subjected to 100 bp single-end sequencing using the Illumina HiSeq 2500 system at the Wayne State University Applied Genomics Technology Center. The sequencing reads were mapped to the human reference genome, and the differential gene expression was determined.

To determine the mRNA expression using quantitative reverse transcription-polymerase chain reaction (qRT-PCR), cDNA libraries were constructed by random priming using the SuperScript III First-Strand Synthesis System (Invitrogen). The synthesized cDNA was used as a template for qRT-PCR with SYBR Green qPCR Master Mixes (Thermo Scientific) on an Mx3000P cycler (Stratagene). All mRNA levels were determined as the delta-delta threshold cycle (ΔΔC_*T*_), and normalized to PPIA mRNA level of PPIA or β-actin housekeeping gene. The results were expressed as the fold changes relative to the untreated control, and the standard deviation (SD) bar graphs were plotted. The PCR primers used are listed in Table S1.

### Dual luciferase reporter assay

Firefly luciferase reporter plasmids (2 μg each) were cotransfected with 0.04 mg of *Renilla reniformis* luciferase control vector Prl-CMV (Promega) into 293FT or A549-Snail cells using Lipofectamine 2000 (Invitrogen). The levels of Snail proteins were increased either by cotransfection of the 293FT cells with pcDNA_3.1_-Snail-HA or pcDNA_3.1_-SnailDN7-HA vector, or treatment of A549-Snail cells with doxycycline. Dual luciferase assays were performed according to the manufacturer’s instructions (Promega).

### Chromatin immunoprecipitation

A549-Snail cells were treated with doxycycline to induce the expression of Snail-HA protein for 2 days, and were then cross-linked in formaldehyde. The nuclei were isolated and sonicated using conditions resulting in prominent DNA fragments between 500 and 750 bp. Chromatin samples were immunoprecipitated with anti-HA antibody (Santa Cruz) and Protein G sepharose (GE Healthcare). DNA samples purified from the precipitated chromatin were used as PCR template. Primers for PCR reactions were: GLDC promoter: 5’-GCTGGGAAGAGGGTAGGAAG-3’ (forward) and 5’-GCGCTCAACCAAGACACTC-3’ (reverse); CDH1 promoter: 5’-GTGAACCCTCAGCCAATCAG-3’ (forward) and 5’-TCACAGGTGCTTTGCAGTTC-3’ (reverse); and GAPDH promoter: 5’-CGGCTACTAGCGGTTTTACG-3’ (forward) and 5’-GCTGCGGGCTCAATTTATAG-3’.

### Statistical analysis

Statistical analyses were performed with GraphPad Prism 6 software (GraphPad Software, La Jolla, CA, USA). Data are presented as means ± SD statistical significance between two groups was determined by unpaired *t*-test.

## Results

### Overexpression of Snail induces epithelial‑to‑mesenchymal transition in A549 cells

**Figure 1:**
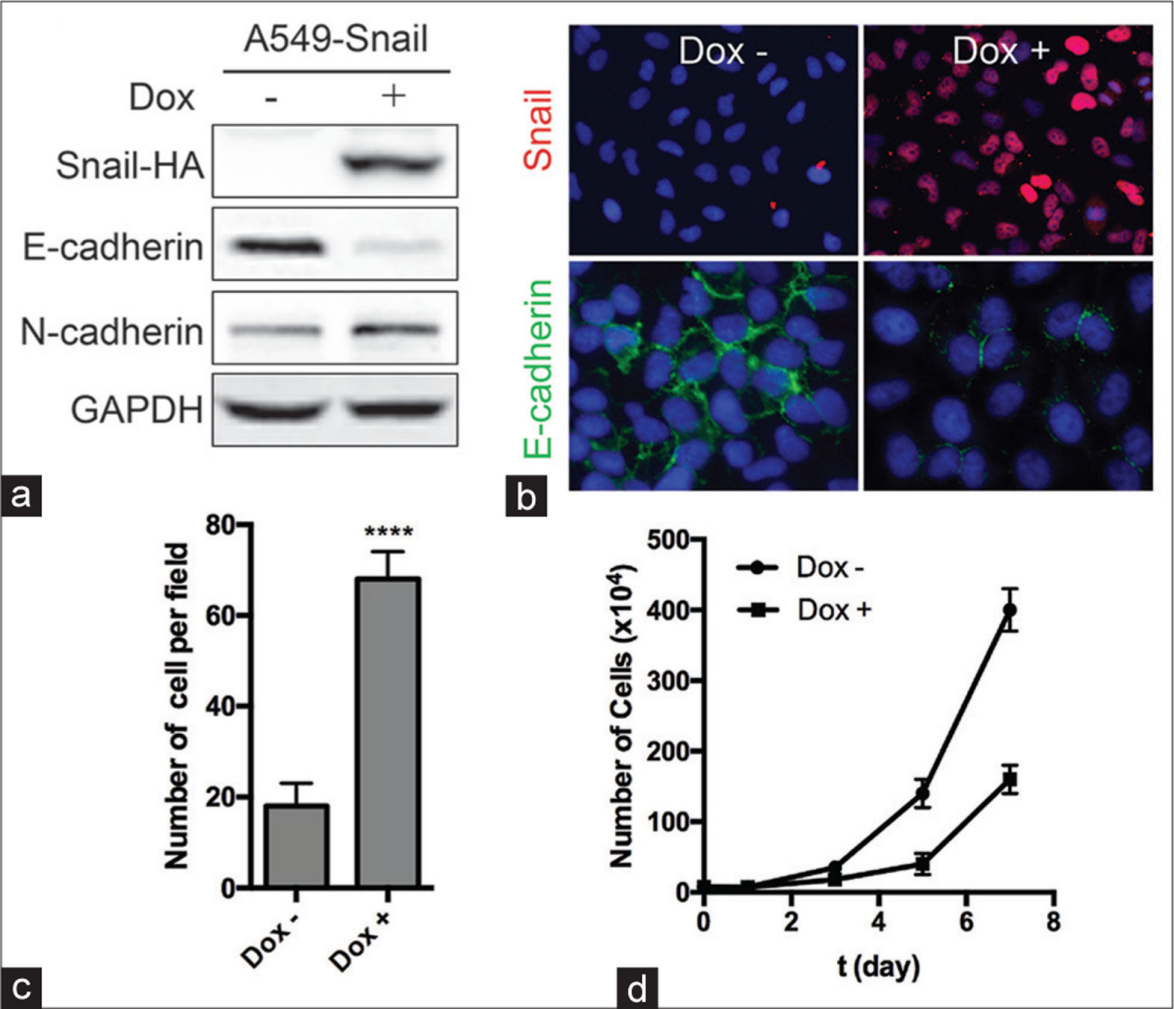
Overexpression of Snail induces the epithelial–mesenchymal transition in A549 cells. A549‑Snail cells, engineered to stably express Snail‑HA under the control of a doxycycline (Dox)‑inducible promoter, were grown in the absence or presence of 2 μg/ml Dox for 2 days. (a) The levels of Snail‑HA, E‑cadherin, N‑cadherin, and GAPDH proteins were measured using Western blotting. (b) The levels of Snail‑HA (red) and E‑cadherin (green) were visualized using immunohistochemistry, and the nuclei were visualized using 4′,6‑diamidino‑2‑phenylindole staining (blue). (c) The number of migratory cells was measured using Boyden chamber assay. ****P < 0.0001 (*t*‑test). (d) A549‑Snail cells were grown in the absence or presence of 2 μg/ml Dox for the indicated time, and the growth kinetics of the cells were measured using cell counting. ****P < 0.0001 (*t*‑test)

To recapitulate the EMT process in tissue culture, we engineered the lentiviral-mediated, doxycycline (Dox)-inducible overexpression of Snail in the A549 cells, a lung carcinoma cell line that has been widely used as *in vitro* model for EMT.^[25]^ As shown in Figure 1, Dox treatment of the stable infectants, designated as the A549-Snail cells, strongly induced the expression of Snail protein in the nuclei, as examined by the Western blotting and immunohistochemistry. Importantly, Snail induction was accompanied by a concomitant reduction and elevation of E- and N-cadherin, the canonical epithelial and mesenchymal gene marker, respectively [Figure 1a and b]. In addition, analyses of cell migratory capacity and growth kinetics demonstrated that Snail induction significantly increased cell migration [Figure 1c] and decreased cell proliferation [Figure 1d]. Thus, the A549-Snail cells exhibit the key characteristics of EMT on Snail induction.

### Overexpression of Snail reprograms cellular metabolic backbone

**Figure 2:**
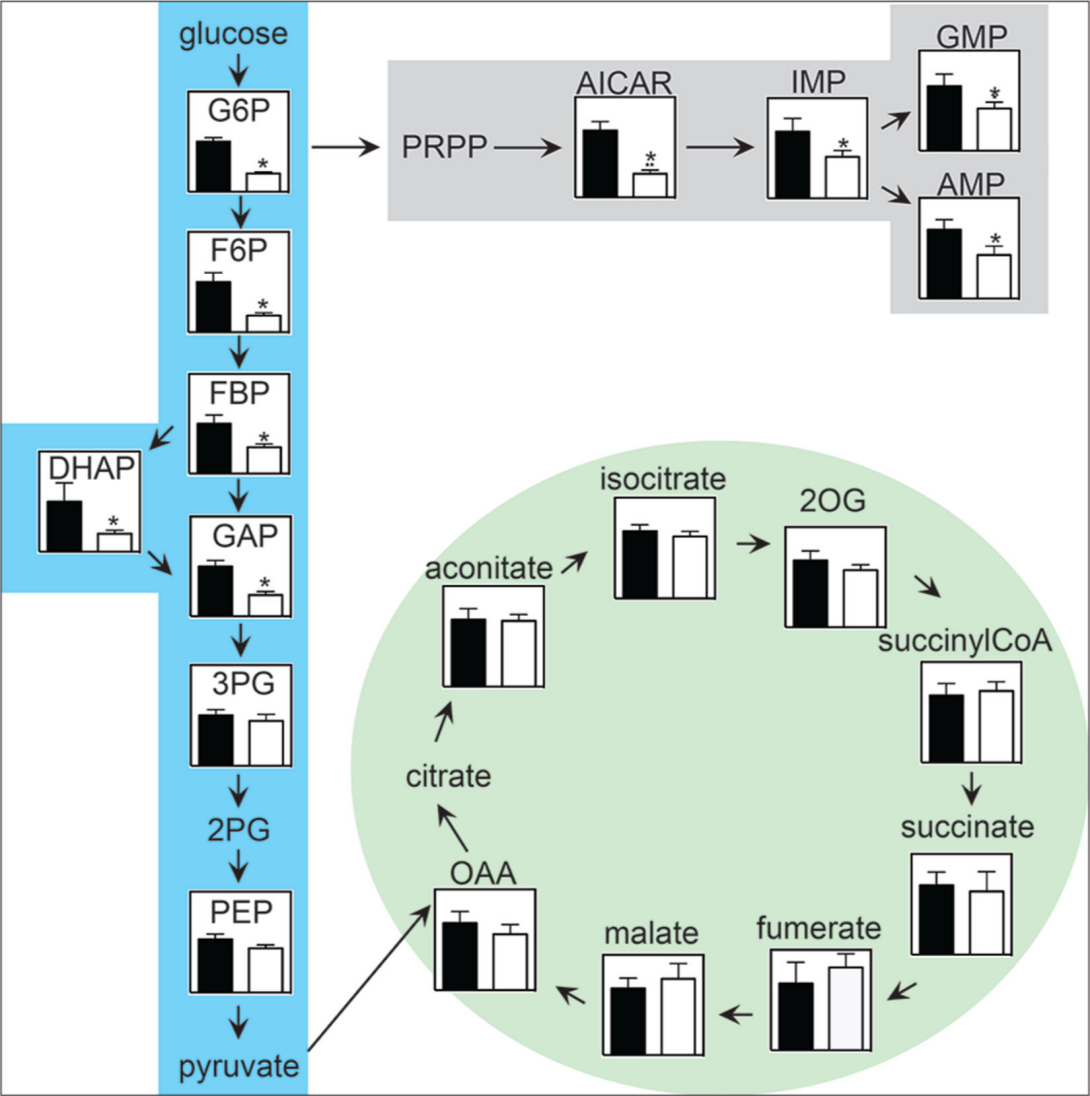
Overexpression of Snail alters the fluctuations of the metabolites on the central metabolic pathways in A549 cells. A549‑Snail cells were grown in the absence (black bars) or presence (white bars) of 2 μg/ml Dox for 2 days. The levels of the metabolites of glycolysis (blue shaded), tricarboxylic acid cycle (green shaded), and nucleotide synthesis (gray shaded) were measured using liquid chromatography‑coupled tandem mass spectrometry/mass spectrometry analysis. *P < 0.05 (*t*‑test)

Wondering if Snail induction triggers metabolic alteration, we measured the levels of numerous metabolites in the A549-Snail cells using liquid chromatography-coupled tandem mass spectrometry (LC-MS/MS) analysis. We observed that Snail induction reduced the levels of the nucleotide biosynthetic intermediates [Figure 2, gray shades], suggesting that the metabolic demand for DNA replication is likely reduced when the proliferation is slowed in the Snail-overexpressing cells [Figure 1d]. Warburg effect denotes a prominent cancerous metabolic phenotype where proliferative cancer cells prefer partial catabolism of glucose in the cytoplasm to complete oxidation of the glucose carbon in the mitochondria.^[26]^ We observed that Snail induction had negligible effects on the levels of the metabolites of the tricarboxylic acid (TCA) cycle [Figure 2, green shades], suggesting that mitochondrial oxidation likely remains as a significant bioenergetics source in support of the cellular commitment to EMT. Interestingly, Snail induction imposed a biphasic change on the fluctuation of the glycolytic metabolites: dividing between glyceraldehyde 3-phosphate (GAP) and 3-phosphoglyceric acid (3PG), Snail induction significantly reduced the levels of the upstream glycolytic intermediates but had negligible effects on the levels of the downstream metabolites [Figure 2, blue shades]. These suggest that distribution of glycolytic metabolites to the downstream metabolic branches, particularly to the one connecting at the junction with GAP/3PG, is dramatically reprogrammed.

### Snail represses the expression of glycine decarboxylase

**Figure 3:**
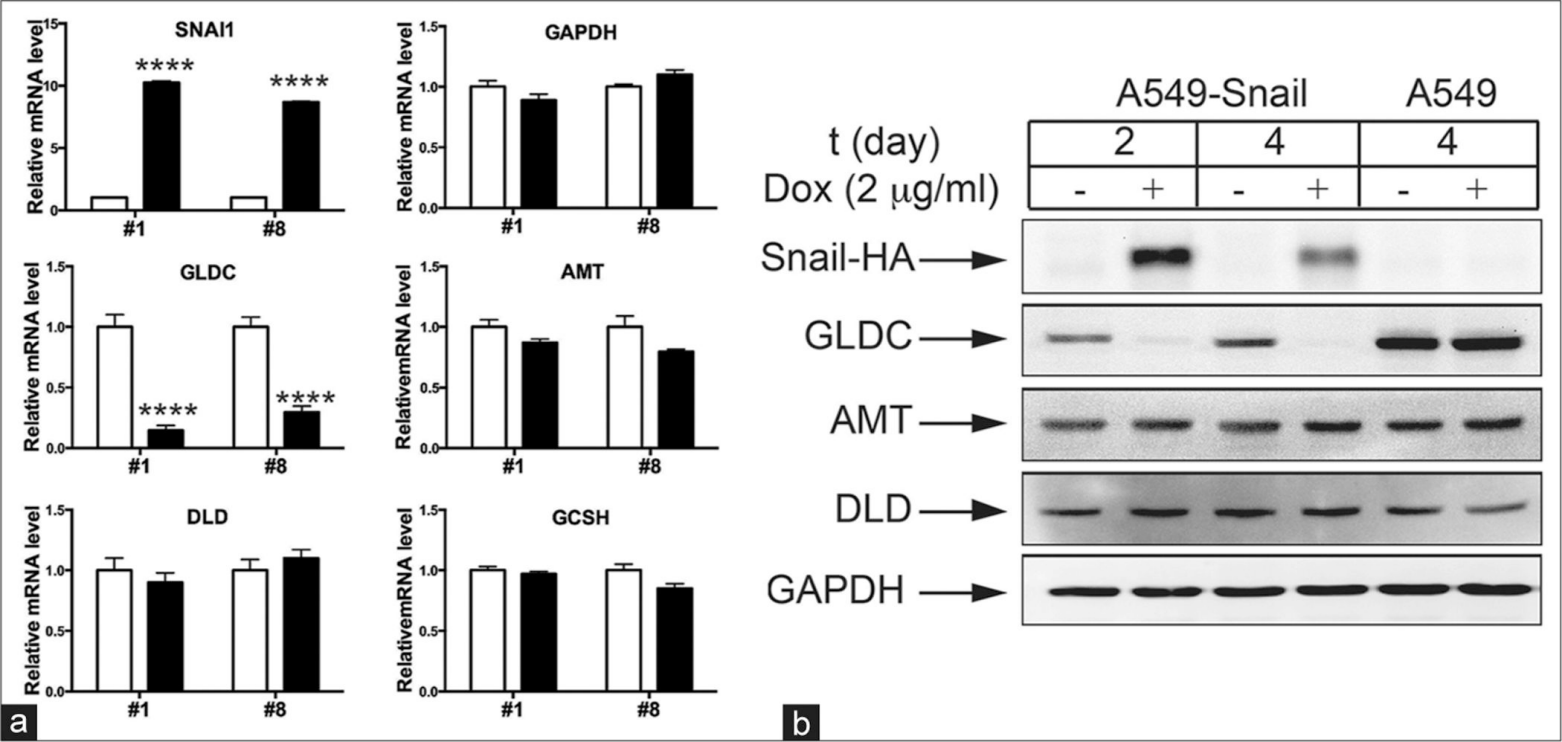
Snail selectively suppresses the expression of glycine decarboxylase. (a) Two clones of A549‑Snail cells (clone #1 and #8) were grown in the absence (white bars) or presence (black bars) of 2 μg/ml Dox for 2 days. The levels of SNAI1, GAPDH, glycine decarboxylase, AMT, DLD, and GCSH mRNAs were measured using quantitative reverse transcription‑polymerase chain reaction. The result is expressed as fold change relative to the level of the uninduced control. (b) A549‑Snail or A549 parent cells were grown in the absence or presence of 2 μg/ml Dox for the indicated time. The levels of Snail‑HA, glycine decarboxylase, AMT, DLD, and GAPDH proteins were measured using Western blotting. ****P < 0.0001 (*t*‑test)

We then investigated the molecular basis for which Snail induces metabolic changes. As Snail is a transcription repressor, we performed gene expression profiling to identify the metabolic enzyme transcripts that decreased in the Snail-overexpressing cells. We found that the transcript encoding GLDC was among the top hits that decreased in response to Snail induction in the A549-Snail cells. GLDC is the rate-limiting component of the glycine cleavage system (GCS), a mitochondrial multimeric enzyme complex that breaks down glycine and concomitantly synthesizes a one-carbon unit, which is a crucial anabolic intermediate required for cell proliferation.^[27]^ As GLDC is a metabolic oncogene previously reported in lung cancer,^[19]^ and it locates on the serine/glycine metabolic shunt that diverts the glycolytic metabolites to the major anabolic pathways,^[13]^ we characterized how Snail regulates the expression of GLDC in detail. We first examined the effects of Snail overexpression on the levels of the transcripts of each GCS components in two clones of the A549-Snail cells. We observed that overexpression of Snail significantly reduced the level of the GLDC transcript without affecting those of the other GCS components including AMT, GCSH, and DLD indicating that Snail selectively suppresses GLDC, the rate-limiting component of GCS [Figure 3a]. Consistently, a similar selective downregulation of GLDC at the protein level was also observed in the Snail-overexpressing cells and was not in the Dox-treated A549 parent cells, which helped to exclude the possibility of which Dox influences the expression of GLDC [Figure 3b]. Thus, our data demonstrated that Snail is a novel suppressor of the *GLDC* gene in lung cancer cells.

**Figure 4:**
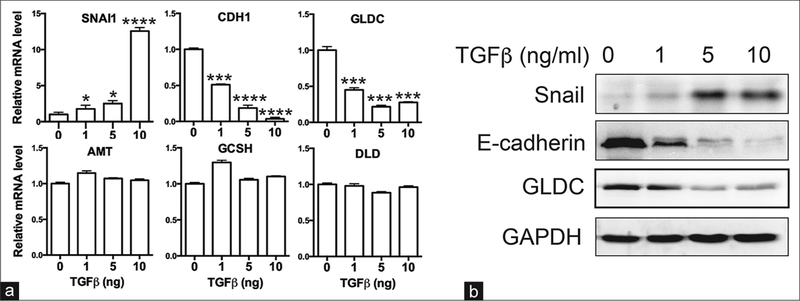
Transforming growth factor‑β treatment led to a concomitant upregulation of Snail and downregulation of glycine decarboxylase expression in A549 cells. A549 cells were treated with the indicated amounts of transforming growth factor‑β for overnight. (a) The levels of SNAI1, CDH1, glycine decarboxylase, AMT, GCSH, and DLD mRNAs were measured using quantitative reverse transcription‑polymerase chain reaction. The result is expressed as fold change relative to the level of untreated control. *P < 0.05, ***P < 0.001, ****P < 0.0001 (*t*‑test). (b) The levels of Snail, E‑cadherin, glycine decarboxylase, and GAPDH proteins were measured using Western blotting

Transforming Growth Factor (TGF)-β is a cytokine that actively induces Snail and thus promotes EMT in A549 cells.^[25]^ We, therefore, tested whether an inverse relationship between Snail and GLDC expression exists in the cells exposed to TGF-β. As expected, treating the cells with increasing amounts of TGF-β led to concomitant upregulation of Snail and downregulation of GLDC at both the transcript and protein levels in dose-dependent manners [Figure 4]. As cytokine signaling conveys the communication between tumor cells and microenvironment, these observations suggest that the newly identified TGF-β/Snail/GLDC axis may be necessary for tumor cells to adapt to the fluctuation of their microenvironment.

### Snail binds to an evolutionarily conserved E‑box motif and thereby inhibits the promoter of glycine decarboxylase

**Figure 5:**
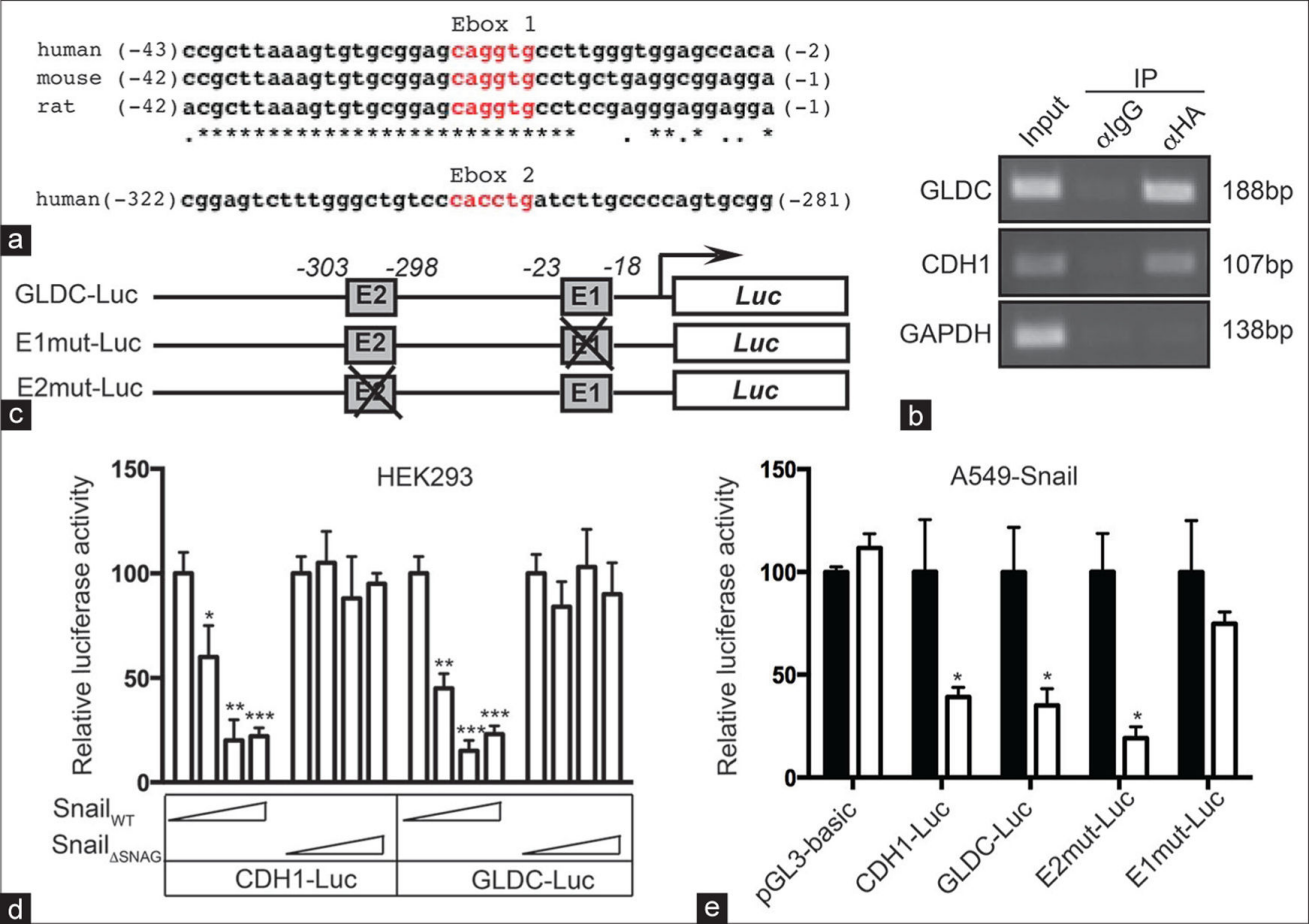
Snail suppresses the promoter of glycine decarboxylase gene by binding to an evolutionarily conserved proximal E‑box motif. (a) glycine decarboxylase genomic sequences at the nucleotide positions from −43 to −2 (top, aligned with the indicated rodent sequences) and from −322 to −281 are exhibited. The E‑box motifs are highlighted in red. (b) A549‑Snail cells were grown in the presence of 2 μg/ml Dox for 2 days. The binding of Snail‑HA to the promoters of glycine decarboxylase, CDH1, and GAPDH were measured using chromatin immunoprecipitation with the anti‑HA antibody followed by polymerase chain reaction amplification with the gene‑specific primers. The amplified products were visualized using agarose gel electrophoresis, and the sizes of each individual amplicon are labeled at the right. (c) A schematic representation of the luciferase reporter constructs. (d) HEK293 cells were transfected with the indicated plasmids for 2 days, and the luciferase activities of the cells were measured. *P < 0.05, **P < 0.01, ***P < 0.001 (*t*‑test). (e) A549‑Snail cells were transfected with the indicated reporter constructs and then grown in the absence (black bars) and presence (white bars) of 2 μg/ml Dox for 2 days, and the luciferase activities of the cells were subsequently measured. *P < 0.05 (*t*‑test)

We then investigated the molecular mechanism of how Snail represses the expression of *GLDC*. As Snail belongs to a family of sequence-specific transcription repressor that recognizes the E-box motif with a consensus of 5’-CAGGTG-3’,^[5,6]^ we searched for the possible Snail binding site(s) in the sequence of human *GLDC* gene. We found two perfectly matched E-box motifs in the promoter of *GLDC* that located at nucleotide positions −23 to −18 and −303 to −298, respectively, relative to the transcription start site. Multi-sequence alignment demonstrated that the proximal motif (positioned at −23 to −18) but not the distal one (positioned at −303 to −298) conserved in two rodent species [Figure 5a]. To test whether Snail interacts with these E-box motifs *in vivo*, we induced the expression of the Snail-HA protein in A549-Snail cells and performed the chromatin immunoprecipitation (ChIP) assay using anti-HA antibody. The ChIP analyses demonstrated that Snail interacted with the promoter of *GLDC* as well as the promoter of *CDH1*, a *bona fide* Snail target, and not with the promoter of *GAPDH*, an irrelevant negative control gene [Figure 5b]. As the interspace of the two *GLDC* E-box motifs only has 278 nucleotides that were not separable in the ChIP assay, we conclude that Snail interacts with at least one of the E-box motifs in the promoter of *GLDC* in A549 cells.

To examine whether and how Snail represses the activity of *GLDC* promoter, we generated a set of luciferase reporters that carry either the wild-type or the mutated *GLDC* promoter [Figure 5c]. The luciferase reporter assays demonstrated that Snail, but not the transcriptionally inactive Snail_ΔSNAG_,^[21]^ inhibited the promoter of *GLDC* as well as the promoter of *CDH1* in a dose-dependent manner [Figure 5d]. In addition, mutagenesis analysis demonstrated that deletion of the proximal but not the distal E-box motif abolished the response of the *GLDC* promoter to Snail [Figure 5e]. Together, our studies identified a proximal E-box motif that mediates the binding and thereby the inhibition of Snail to the promoter of *GLDC*.

### Knockdown of glycine decarboxylase inhibits the proliferation and promotes the migration of A549 cells

**Figure 6:**
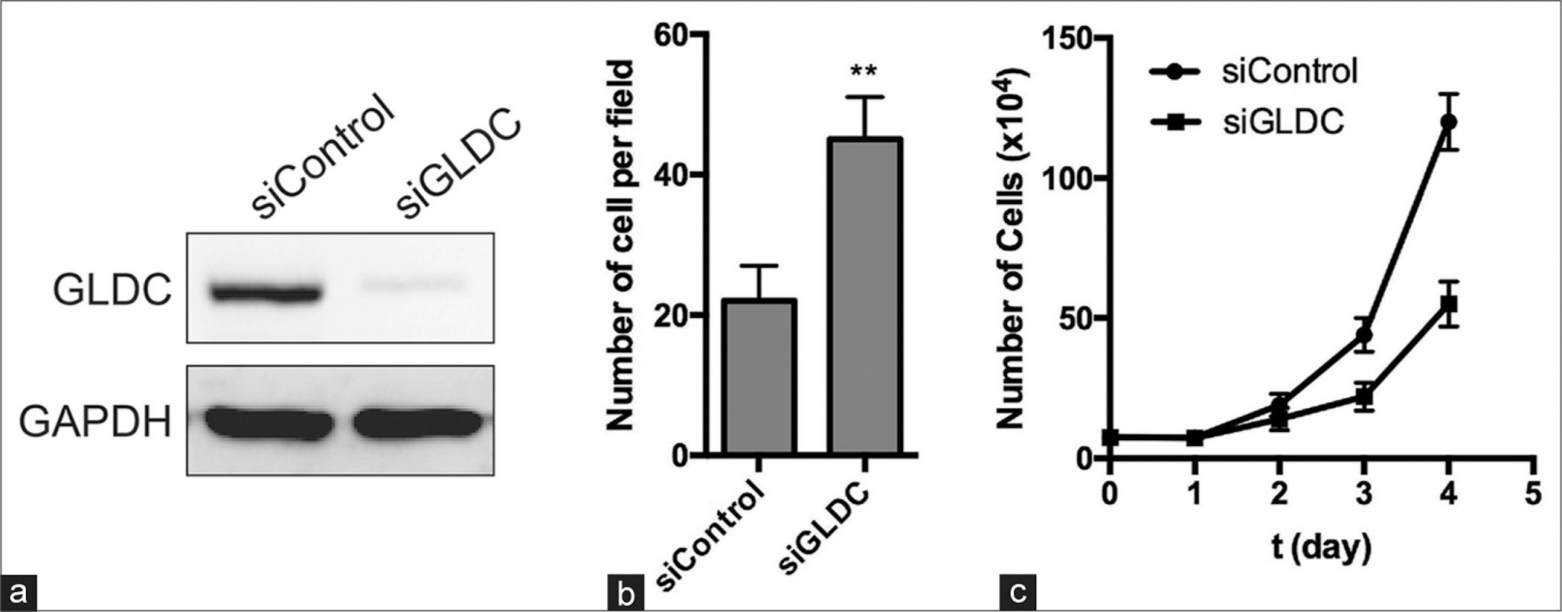
Knockdown of glycine decarboxylase induces a shift of cellular function from proliferation to migration in A549 cells. A549 cells were transfected with either the scrambled (siControl) or glycine decarboxylase‑specific siRNA oligos for 2 days. (a) The levels of glycine decarboxylase and GAPDH proteins were measured using Western blotting. (b) The number of migratory cells was measured using Boyden chamber assay. **P < 0.01 (*t*‑test). (c) A549 cells were transfected with either the siControl or siGLDC oligos, and the growth kinetics of the transfected cells were measured using cell counting within the indicated time frame. **P < 0.01 (*t*‑test)

To examine how reduced expression of GLDC influences the cell behavior, we knocked down GLDC in A549 cells using small interference RNA (siRNA). The data demonstrated that transfection of the siRNA oligos specific for *GLDC* reduced the expression of GLDC protein by ~ 80%, and significantly decreased the proliferation and increased the migration of A549 cells [Figure 6]. Thus, reducing GLDC expression mimics the effects of Snail overexpression on the proliferation and migration of A549 cells.

## Discussion

As metabolic reprogramming and cellular plasticity are common features for the adaption of cells to the environment,^[7–9]^ it is interesting to address whether these events are functionally coordinated. Concerning to tumor biology, it is mechanistically insightful and therapeutically useful to elucidate how metabolic reprogramming couples with the EMT program, which could help for developing novel therapeutic approaches against metastasis and chemoresistance through disrupting the metabolic basis for tumor plasticity.^[28,29]^ In this study, we examined how gain of Snail, a prototypic EMT transcription factor and an oncogenic protein, affects the fluctuation of the backbone metabolic pathways in lung cancer cells, and further identified that GLDC is a novel metabolic component integral to the Snail-induced EMT program.

To robustly examine the metabolic fluctuation in the process of EMT, we established an inducible EMT tissue culture model through ectopic expression of Snail under the control of doxycycline-inducible promoter in the lung cancer A549 cells. We demonstrated that induction of Snail robustly activated EMT, as manifested by the appearance of the critical molecular and functional signatures of EMT, including concomitant reduction of E-cadherin and elevation of N-cadherin, as well as increase of migration and decrease of proliferation in the Snail-expressing cells. Using targeted metabolomics analysis, we probed the influence of Snail overexpression on the metabolite fluctuation of the backbone metabolic pathways, those including nucleotide synthesis, glycolysis, and TCA cycle. We observed that the levels of nucleotide biosynthetic intermediates were uniformly decreased in the Snail-expressing cells, suggesting the metabolic demand for DNA synthesis possibly decreased when the cells commit to EMT and slow down proliferation. In contrast, the levels of TCA cycle metabolites remained unchanged in the Snail-expressing cells, indicating that mitochondrial oxidation is likely bioenergetically necessary for supporting the EMT-committed cells. Interestingly, a biphasic response in the levels of the glycolytic metabolites to the Snail induction was observed. After Snail induction, dividing at the interface between GAP and 3PG, the levels of the upstream glycolytic metabolites were decreased, yet the levels of the downstream ones remained unchanged. At present, we do not fully understand the logic behind this clear split in the alterations of the glycolytic metabolites in response to Snail induction. However, a clue may lie in the fact that in addition to feeding the TCA cycle, glycolysis diverts its metabolic flux to specific key branching pathways including the pentose phosphate pathway and the serine/glycine metabolic shunt.^[13]^ We predict that the sharp divergence of the alterations of the glycolytic metabolites under the condition of Snail induction may reflect a significant rearrangement of a metabolic branch that connects with the glycolytic pathway at the interface of GAP and 3PG. Indeed, the serine/glycine metabolic shunt, a vital connector between glycolysis and key anabolic pathways, is right started using 3PG as metabolic substrate.^[13]^ Thus, our metabolite analysis predicted that Snail significantly rearranges the serine/glycine metabolism in the EMT commitment of the lung cancer cells.

Interestingly, our gene expression profiling identified that the expression of GLDC, a key enzyme of the serine/glycine metabolic branch was significantly reduced in response to Snail induction. Our molecular characterization further defined that an evolutionarily conserved proximal E-box motif in *GLDC* gene mediates the binding and thereby the inhibition of Snail to the *GLDC* promoter. Also, we demonstrated that treatment of cells with TGFβ, a canonical EMT-inducing cytokine, led to concomitant induction of Snail and suppression of GLDC in dose-dependent manners. Thus, we demonstrated that this newly identified Snail/GLDC axis is an integral component of the EMT program. GLDC is the rate-limiting enzyme of GCS that catabolizes glycine for the synthesis of critical anabolic substrate – the folate-mediated one-carbon unit.^[27]^ GLDC is a metabolic oncogene sufficient to drive the proliferation of cancer cells.^[19]^ For metabolic networking, GLDC, as a bottleneck enzyme, is essential for cancer cells to sustain the metabolic flux through the serine/glycine shunt.^[30]^ In this sense, it is plausible that GLDC functions as a regulatory checkpoint that influences the activity of the serine/glycine branch and in turn, the distribution of glycolytic flux in response to Snail induction. Indeed, the functional significance of this newly identified Snail/GLDC axis is further supported by the observation that reduction of GLDC is sufficient to induce a switch of cellular phenotype from proliferation to migration in both the present [Figure 6] and previous^[31]^ studies.

In summary, the present study has identified that GLDC is a novel metabolic target directly controlled by Snail. It further suggests that the Snail/GLDC regulatory axis, as an integral part of the EMT program, poses a significant impact on the diversion of glycolytic flux to biosynthetic pathways, and thereby the arrangement of global anabolic/catabolic activities in the process of EMT. However, the functional importance of this novel regulatory link in the context of tumor biology needs further investigation.

## Supplementary Material

1
