# Supplementary material for: Identification and Characterization of Glycine Decarboxylase as a Direct Target of Snail in the Epithelial–Mesenchymal Transition of Cancer Cells
Source: Tumor Microenviron. Author manuscript; Available in PMC 2022 Apr 1. (PMC8049539; doi:10.4103/tme.tme_8_18)
Supplement: 1 [file NIHMS1061557-supplement-1.pdf]

Supplement Table

Table S1: Quantitative polymerase chain reaction primer for human gene

Gene	Forward sequence	Reverse sequence
GLDC	ACCAATGGGGTGTTTGAAGA	CAGATTCCCACCTGAGCATT
AMT	TGGTTGGAGACATTGCAGAG	AGCGTTGGACACCACATACA
GCDH	CTCCCTGAAGTTGGGACAAA	CCTGGATTTTCTGCAAGAGC
DLD	GGTTGGCAAATCAGAAGAGC	GGATCTTCACCATGCCATCT
CDH1	TGGACAGGGAGGATTTTGAG	GGCGTTGTCATTCACATCAG
CDH2	TGCACAGATGTGGACAGGAT	CCACAAACATCAGCACAAGG
SNAI1	CTCTAGGCCCTGGCTGCTAC	TGACATCTGAGTGGGTCTGG
SNAI2	TTCGGACCCACACATTACCT	GCAGTGAGGGCAAGAAAAAG
GAPDH	CTCTGCTCCTCCTGTTCGAC	GCGCCCAATACGACCAAATC

GLDC: Glycine decarboxylase
